# Constitutive expression of *REL1* confers the rice response to drought stress and abscisic acid

**DOI:** 10.1186/s12284-018-0251-0

**Published:** 2018-10-25

**Authors:** Jiayan Liang, Shaoying Guo, Bo Sun, Qing Liu, Xionghui Chen, Haifeng Peng, Zemin Zhang, Qingjun Xie

**Affiliations:** State Key Laboratory for Conservation and Utilization of Subtropical Agro-Bioresources, Guangdong Provincial Key Laboratory of Plant Molecular Breeding, South China Agricultural University, Guangzhou, 510642 China

**Keywords:** Rice, *REL1*, Leaf morphology, Genomics, ABA, Stress response

## Abstract

Leaf rolling is one of the most significant symptoms of drought stress in plant. Previously, we identified a dominant negative mutant, termed *rolled and erect 1* (hereafter referred to *rel1-D*), regulating leaf rolling and erectness in rice. However, the role of *REL1* in drought response is still poorly understood. Here, our results indicated that *rel1*-*D* displayed higher tolerance to drought relative to wild type, and the activity of superoxide dismutase (SOD) and drought responsive genes were significantly up-regulated in *rel1-D*. Moreover, our results revealed that *rel1-D* was hypersensitive to ABA and the expression of ABA associated genes was significantly increased in *rel1-D*, suggesting that *REL1* likely coordinates ABA to regulate drought response. Using the RNA-seq approach, we identified a large group of differentially expressed genes that regulate stimuli and stresses response. Consistently, we also found that constitutive expression of *REL1* alters the expression of biotic and abiotic stress responsive genes by the isobaric tags for relative and absolute quantification (iTRAQ) analysis. Integrative analysis demonstrated that 8 genes/proteins identified by both RNA-seq and iTRAQ would be the potential targets in term of the *REL1-*mediated leaf morphology. Together, we proposed that leaf rolling and drought tolerance of *rel1-D* under normal condition might be caused by the endogenously perturbed homeostasis derived from continuous stressful dynamics.

**Electronic supplementary material:**

The online version of this article (10.1186/s12284-018-0251-0) contains supplementary material, which is available to authorized users.

## Background

Crop yield is adversely challenged by drought stress, one of the most major environmental stresses, of which the occurrence and severity are both increased due to the recent climate change, inadequate water supply and world population growth worldwide. Therefore, improving drought tolerance of crop is an important objective to overcome such issue and provide enough world food (Yamaguchi-Shinozaki and Shinozaki, 2006). One of the most significant symptoms of drought stress in plant is the leaf rolling. Plant leaf generally is polarized along the adaxial-abaxial axis (Itoh et al. 2005), thereby generating two types of leaf forms under unfavorable conditions: abaxially leaf rolling and adaxially leaf rolling. Moderate leaf rolling promotes rice yield by increasing the photosynthetic efficiency and reducing the transpiration (Lang et al. 2004; Zhang et al. 2009; Zou et al. 2011). In addition, moderate leaf rolling also facilitates the survival and development of plant under stress conditions (Kadioglu et al. 2012). Therefore, manipulation of adaxial and abaxial leaf rolling would be one of the most important strategies to increase the rice productivity and tolerance to drought and other stresses in coming years (Zou et al. 2011).

Generally, leaf rolling is largely regulated by several factors: hormones (Fujino et al. 2008; Lee et al. 2006), metabolic changes (Talukdar and Talukdar, 2014) and formation of specific cells, such as bulliform cell (Fang et al. 2012; Xiang et al. 2012). To date, more than 30 genes responsible for the rice leaf rolling have been identified. For example, *Abaxially Curled Leaf 1* (*ACL1*), *Abaxially Curled Leaf 2* (*ACL2)* (Li et al. 2010) and *Rice outermost cell-specific gene 5* (*Roc5*) (Zou et al. 2011) are involved in abaxial leaf rolling, whereas *SEMI-ROLLED LEAF 1* (*SRL1*) (Xiang et al. 2012), *ROLLED LEAF 9* (*RL9*)/*SHALLOT-LIKE1* (*SLL1*) (Zhang et al. 2009; Yan et al. 2008), *OsAGO7* (Shi et al. 2007), *Rolling-leaf 14* (*RL14*) (Fang et al. 2012), *R2R3-MYB transcription factor* (*OsMYB103L*) (Yang et al. 2014), *ADAXIALIZED LEAF 1* (*ADL1*) (Hibara et al. 2009) and *Narrow and Rolled Leaves 1* (*NRL1*) (Wu et al. 2010) participates in adaxial leaf rolling. In addition, other regulators, such as *CONSTITUTIVELY WILTED1* (*COW1*)/*NAL7* (Fujino et al. 2008), has also been well-characterized in term of their involvements in leaf rolling. Even thought, it is still poorly understood whether these leaf rolling genes participate in stress response, in particular drought.

To cope with drought stress, plants have been evolved a sophisticated adaptation mechanisms to increase their chance of survival through coordinating the expression of drought responsive genes, which retarded leaf rolling, wilting, and loss of chlorophyll via ABA-dependent or independent pathways (Yamaguchi-Shinozaki and Shinozaki, 2006; Weiner et al. 2010; Cutler et al. 2010). Under drought stress, ABA accumulates in plant cells and stimulates the core ABA signaling transduction through the ABA receptors *PYR/PYL/RCAR*, *Protein Phosphatases 2C* (*PP2Cs*), and subclass III *SNF 1-RELATED PROTEIN KINASE 2* (*SnRK2*) protein kinases (Weiner et al. 2010). For example, loss of the rice subclass-I and -II SnRK2s (*OsSAPK2*) confers the more sensitivity to drought stress and insensitive to ABA (Lou et al. 2017). Over-expression of *OsDT11* enhanced drought tolerance thought the ABA signaling pathway in rice (Li et al. 2017). In contrast, a group of regulators modulate drought tolerance by ABA-independent pathway. Fox example, *OsMADS50* was markedly induced by low water-deficit treatment but not ABA, and the *osmads50* knockout mutant exhibited significant delays in flowering compared with the WT, indicating *OsMADS50* had an ABA-independent and positive role in drought response (Du et al. 2018).

Here, we were particularly interested in elucidating the relationship between the *rel1-D* mediated leaf rolling and drought stress in rice. Our results suggested that up-regulation of *REL1* confers more drought tolerance by eliminating the ROS level and triggers the ABA response. Similarly, transcriptomic and proteomic profiling of *rel1-D* and wild type also revealed that most of the differentially expressed genes (DEGs) and differentially expressed proteins (DEPs) are mainly involved in metabolic changes and stress responses. Collectively, we proposed that leaf rolling of *rel1-D* is caused by the altered dynamics of stress response endogenously.

## Methods

### Plant materials and growth conditions

All plants (wild type and *rel1-D* mutants) used in this study were derived from our previous study (Chen et al. 2015). All rice seeds in this study were propagated in the paddy field in Guangzhou, China. For laboratory work, rice plants were grown in a greenhouse under a16-h-light/8-h-dark cycle at 30 °C. No significant differences were observed when plants were grown in the greenhouse compared to the paddy field.

### Drought response assay

For drought tolerance test, wild type and *rel1-D* seedlings were grown in soil for 2 months, and then treated by withholding water for 14 days, followed by recovery irrigation for 7 days. Leaves from 1-month-old seedling were selected for dark-induced leaf senescence and ABA treatment. Seeds of wild type and *rel1-D* were germinated at 37 °C, and then transferred into 1/2 MS medium for 14 days at 30 °C under a16-h-light/8-h-dark cycle. Seedlings from 3rd leaf stage were selected for PEG treatment: 1) plants treated with 0%, 10%, 20%, 30% 40% PEG4000 for 24 h, and the rolling index was measured. The rolling index was measured as previous study (Shi et al. 2007). 2) Plants treated with 20% PEG4000 with 5 time courses, including 0 h, 3 h, 6 h, 12 h and 24 h. The SOD activity was measured as previous study (Zhang et al. 2005).

### ABA treatment and chlorophyll content measurement

Leaves were treatment with distillation-distillation water with 0 μM or 20 μM ABA at 28 °C in darkness for 5 days. Chlorophyll content was measured as followed: leaves were milled in 95% ethanol; homogenate solution adjusted to 50 ml final volume; measure the absorbance at 652 nm. Calculate the chlorophyll content according to the following formula: chlorophyll content (mg/g) = (A_652_ × V)/ (34.5 × m); V was the final volume of homogenate solution, and m was the weight of leaves.

### RNA extraction and quantitative real-time PCR

Total RNA was extracted using the RNeasy Plant Mini Kit (Qiagen) according to the manufacturer’s instructions. The first strand of cDNA was synthesized using TransScript First-Strand cDNA Synthesis SuperMix (TransGen Biotech) and quantitative real-time polymerase chain reaction (qRT-PCR) was performed as previously described (Chen et al. 2015). The relative expression level of a target gene was normalized to that of rice *UBC*. All primers used in qRT-PCR are listed in Additional file 1: Table S12.

### Sequence reads mapping

Raw data (raw reads) of fastq format were firstly processed through in-house perl scripts. In this step, the clean data (clean reads) were obtained by removing reads containing adapter, reads containing poly-N and low quality reads from raw data. At the same time, quality parameters of clean data including Q20, Q30, GC-content and sequence duplication level were used for data filtering. All the succeeding analyses were carried out using high quality clean data. Reference genome and gene model annotation files were downloaded from The MSU Rice Genome Annotation Project Database website at http://rice.plantbiology.msu.edu. An index of the reference genome was built using Bowtie2 v2.2.5 (Langmead et al. 2009) and paired-end clean reads were aligned to the reference genome using TopHat v2.0.14 (Trapnell et al. 2010). TopHat was chosen as the mapping tool, because it can generate a database of splice junctions based on the gene model annotation file, and thus give a better mapping result than other non-splice mapping tools.

### Quantification and differential expression analysis of transcripts

HTSeq v0.6.1 (http://www-huber.embl.de/users/anders/HTSeq) was used to count the reads numbers mapped to each transcript. The parameter FPKM (expected number of Fragments per kilo-base of transcript sequence per Millions base pairs sequenced) was used to quantify transcripts expression. FPKM was calculated based on the mapped transcript fragments, transcript length and sequencing depth. Currently, this is the most commonly used method for estimating transcript expression (Trapnell et al. 2010). Differential expression analysis of two conditions/groups was performed using the DESeq R package (1.10.1) (Anders and Huber, 2010). DESeq provides statistical routines to determine differential expression in digital gene expression data using a model based on the negative binomial distribution. The resulting *q*-values (*p*-adjusted) were adjusted using the Benjamini and Hochberg’s approach for controlling the false discovery rate (Anders and Huber, 2010). Genes with an adjusted |log_2_ (FC)| ≥ 1 and FDR < 0.05 were assigned as differentially expressed.

### iTRAQ assay and data analysis

The iTRAQ assay was performed by the BGI Company. Briefly, about 100 μg protein was subjected to the LC-ESI-MS/MS analysis based on the Triple TOF 5600. The proteins identification was performed by using Mascot search engine (Matrix Science, London, UK; version 2.3.02). For protein quantitation, it was required that a protein contains at least two unique spectra. The quantitative protein ratios were weighted and normalized by the median ratio in Mascot. We used ratios of *p*-values < 0.05, and fold changes > 1.5 or < 0.67 was considered as significant. Functional annotations of the proteins were conducted using Blast2GO program against the non-redundant protein database (https://www.ncbi.nlm.nih.gov/protein/). The keg database (http://www.genome.jp/kegg/) and the COG database (http://www.ncbi.nlm.nih.gov/COG/) were used to classify and group these identified proteins.

## Results

### Response of *rel1-D* to drought stress

**Fig. 1:**
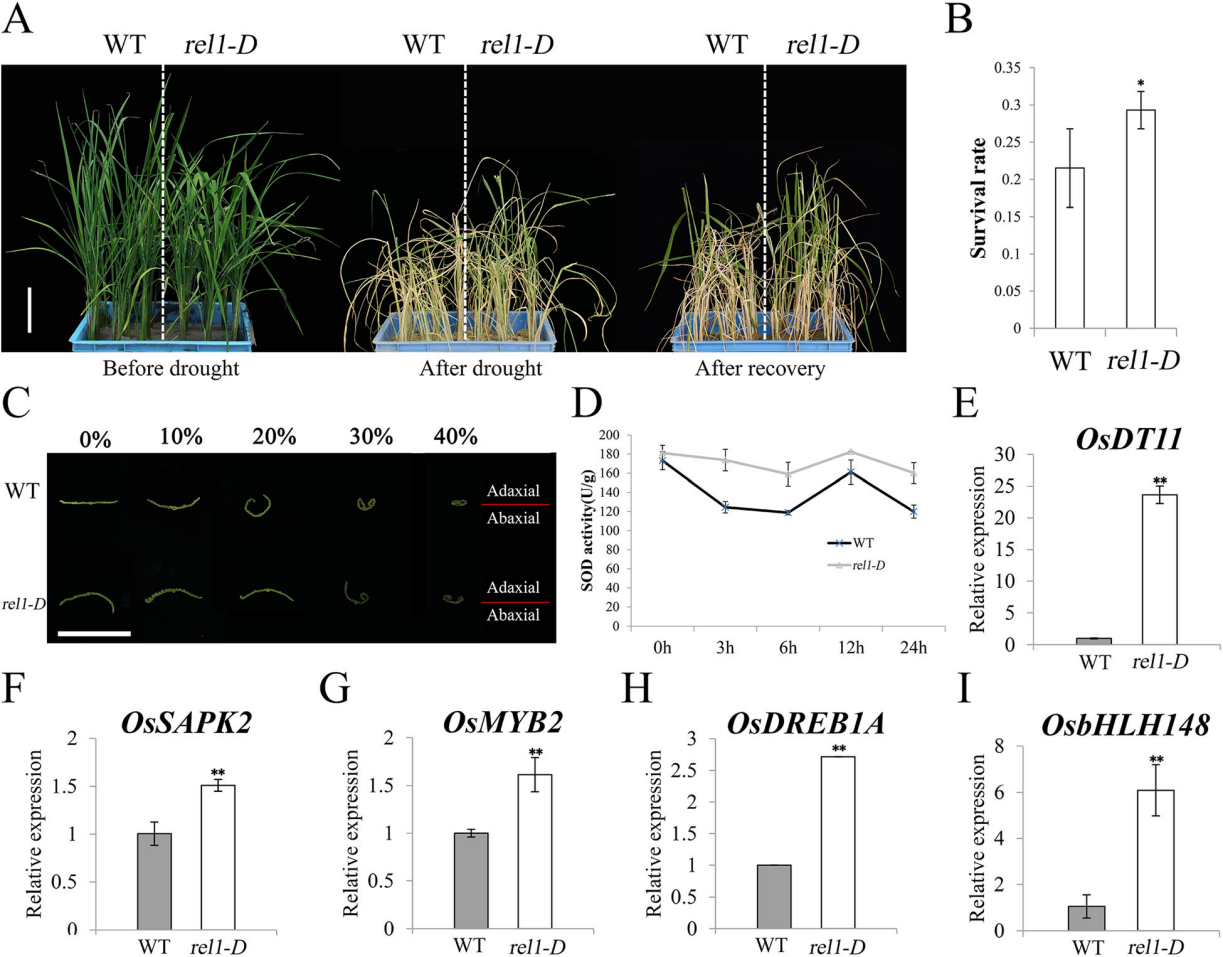
Response of *rel1-D* to drought stress. **a**, Phenotype of WT and *rel1-D* with drought treatment. Two-month-old seedlings were used, bars = 10 cm. **b**, Survival rate of WT and *rel1-D* were derived from the (A). **c**, Third leaf stage seedling of WT and *rel1-D* were treated by different concentration of PEG, bars = 5 mm. **d**, Measurement of SOD activity in wild type and *rel1-D*. E-I, Expression of drought-responsive genes in wild type and *rel1-D*. **b** and **e**-**i**, Data were presented as mean ± S.E., * *p*-value < 0.05, ** *p*-value < 0.01, two-tailed, two sample Student’s t test

Leaf rolling generally leads to reduction of water loss, and thereby enhances tolerance to multiple stresses (Lang et al. 2004; Zhang et al. 2009; Zou et al. 2011). Therefore, we were specifically interested in investigating the involvement of *REL1* in stress response. To address this issue, WT and *rel1-D* seedlings were undergone the drought assay. Phenotypic analysis showed that almost all of the WT plants displayed severe growth retardation and wilting while the *rel1-D* plants exhibited less abnormal phenotypes by withholding irrigation for 14 days, and then the *rel1-D* but not the WT was recovered upon re-watering for 7 days (Fig. 1a and b). To further explore the drought tolerance of *rel1-D*, leaves from WT and *rel1-D* were treated by polyethylene glycol (PEG) 4000 at different concentrations. Our results indicated that leaves of *rel1-D* were much more insensitive to the treatment than those of WT (Fig. 1c). The superoxide dismutase (SOD) activity is generally used as an important indicator for drought tolerance. Therefore, the SOD activity of WT and *rel1-D* that treated by 20% PEG4000 was measured at 5 time courses. Our results demonstrated that the change pattern of SOD activity was similar between WT and *rel1-D*, but the corresponding levels were higher in *rel1-D* than those in WT (Fig. 1d), suggesting that up-regulating *REL1* suppressed the boost of reactive oxygen species (ROS) under drought stress. To further gain insight into the *REL1*-mediated drought resistance, we then evaluated the expression of drought-responsive marker genes. Our results demonstrated that *OsDT11*, *OsSAPK2*, *OsMYB2*, *OsDREB1A* and *OsbHLH148*, functioning as positive regulators in drought response (Lou et al. 2017; Li et al. 2017; Chen et al. 2008; Dubouzet et al. 2003; Seo et al. 2011; Yang et al. 2012), were significantly up-regulated in the *rel1-D* (Fig. 1e-i). Consistently, the similar patterns of above marker genes were also found in the RNA-seq result (Additional file 2: Table S1). These results suggested that enhanced the expression of *REL1* leads to the substantial higher expression of drought-responsive genes endogenously, eventually resulting in the tolerance of *rel1-D* to drought.

Taking into account that *REL1* is an abaxial leaf rolling regulator, we wondered whether other abaxial leaf rolling associated genes is also involved in the response to drought stress. To address this issue, we examined their expression pattern in WT and *rel1-D* at different time courses by PEG treatment. Surprisingly, *REL1* exhibited normal expression during the treatment in WT, implying it is not related with drought stress. However, the expression level of *REL1* was substantial higher in *rel1-D* than that in WT even it was attenuated at 3 h but gradually increased afterward in *rel1-D* (Additional file 3: Figure S1A). The *ACL1* was not changed by the PEG treatment in both WT and *rel1-D* at 24 h (Additional file 3: Figure S1B), suggesting this leaf rolling gene is not involved in drought response. The *ACL2* was significantly enhanced at 24 h in WT, implying it might participate in drought stress. Notably, *ACL2* was retarded from 3 h to 12 h but increased to the normal level at 24 h in *rel1-D* with the similar level of that in WT (Additional file 3: Figure S1C), suggesting that constitutive expression of *REL1* would repress *ACL2* during drought stress. The *Roc5* was down-regulated at 12 h and increased to the normal level at 24 h in WT, but gradually decreased in *rel1-D* during treatment (Additional file 3: Figure S1D), demonstrating *Roc5* was suppressed by up-regulation of *REL1* under drought stress. Taken together, we proposed that *rel1-D* mediated drought tolerance independent on the three leaf rolling genes, but *ACL2* may be also involved in drought stress in a distinct pathway.

### Response of *rel1-D* to ABA

**Fig. 2:**
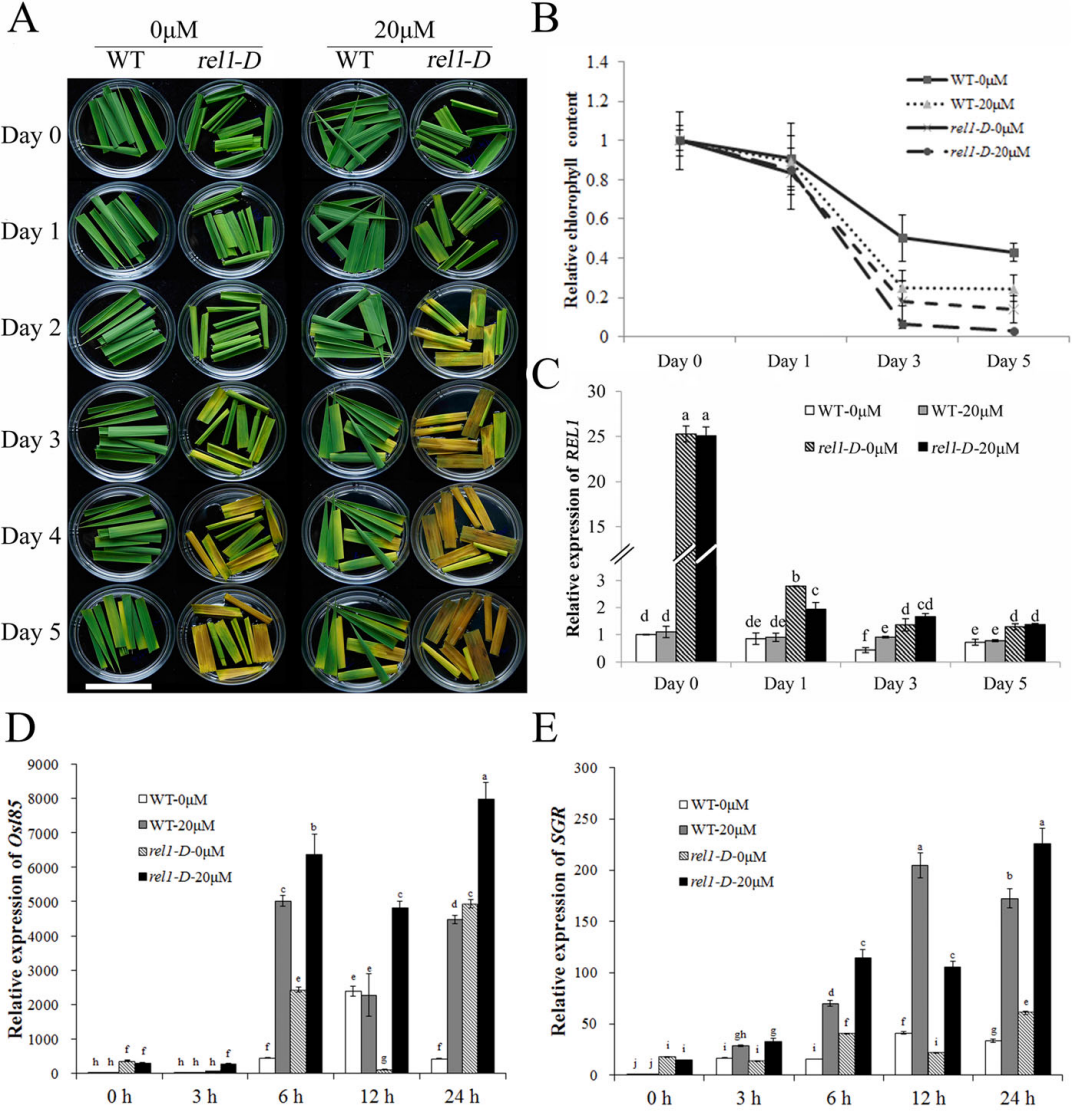
Response of *rel1-D* to ABA. **a**, Response of *rel1-D* to ABA during dark-induced leaf senescence. Leaves from 1-month-old seedling of WT and *rel1-D* were incubated with 20 μM ABA for 1 to 5 days, bars = 5 cm. **b**, Chlorophyll content of WT and *rel1-D* by ABA treatments. **c**, Expression of *REL1* in response to ABA treatment. **d**, Expression of *Osl85* in response to ABA treatment. **e**, Expression of *SGR* in response to ABA treatment. B-E, Data were presented as mean ± S.E.. **c**-**e**, Multiple comparisons, Duncan, *p*-value < 0.01

Since ABA both regulates drought response and leaf senescence (Lou et al. 2017; Li et al. 2017; Chen et al. 2008; Dubouzet et al. 2003; Seo et al. 2011; Yang et al. 2012), we wondered whether *REL1* was also involved in the response to ABA. To address this issue, leaves from WT and *rel1-D* were treated by 0 and 20 μM ABA from 1 to 5 days by the dark-induced leaf senescence assay, respectively. Under the ABA treatment, *rel1-D* displayed early senescence phenotype and rapid degradation of chlorophyll (Chl) rather than that in WT (Fig. 2a and b), indicating it was hypersensitive to ABA. Notably, *REL1* is significantly repressed by the ABA treatments (Fig. 2c). Therefore, we concluded that boosting *REL1* accelerates ABA-induced leaf senescence. It was worthy to figure out that *rel1-D* leaves started to turn yellowish while the wild-type leaves still remained green at the 3 days. Five days after dark treatment, the Chl content in the *rel1-D* leaves was about 2-fold less than in the wild-type leaves (Fig. 2a and b). To further explore the relationship between *REL1* and ABA-induced senescence, we detected the expression of the senescence marker genes *Osl85* and *SGR*, and found that they were induced by dark-treatment assay as previously reported (Lee et al. 2001; Jiang et al. 2011). Notably, their levels were significantly higher in *rel1-D* as compared to WT, as well as with ABA rather than without ABA treatment (Fig. 2d and e). Therefore, we proposed that overexpression of *REL1* also triggers the natural leaf senescence and ABA would accelerate this response.

### Transcriptomic profiling of the *rel1-D* mutant

**Fig. 3:**
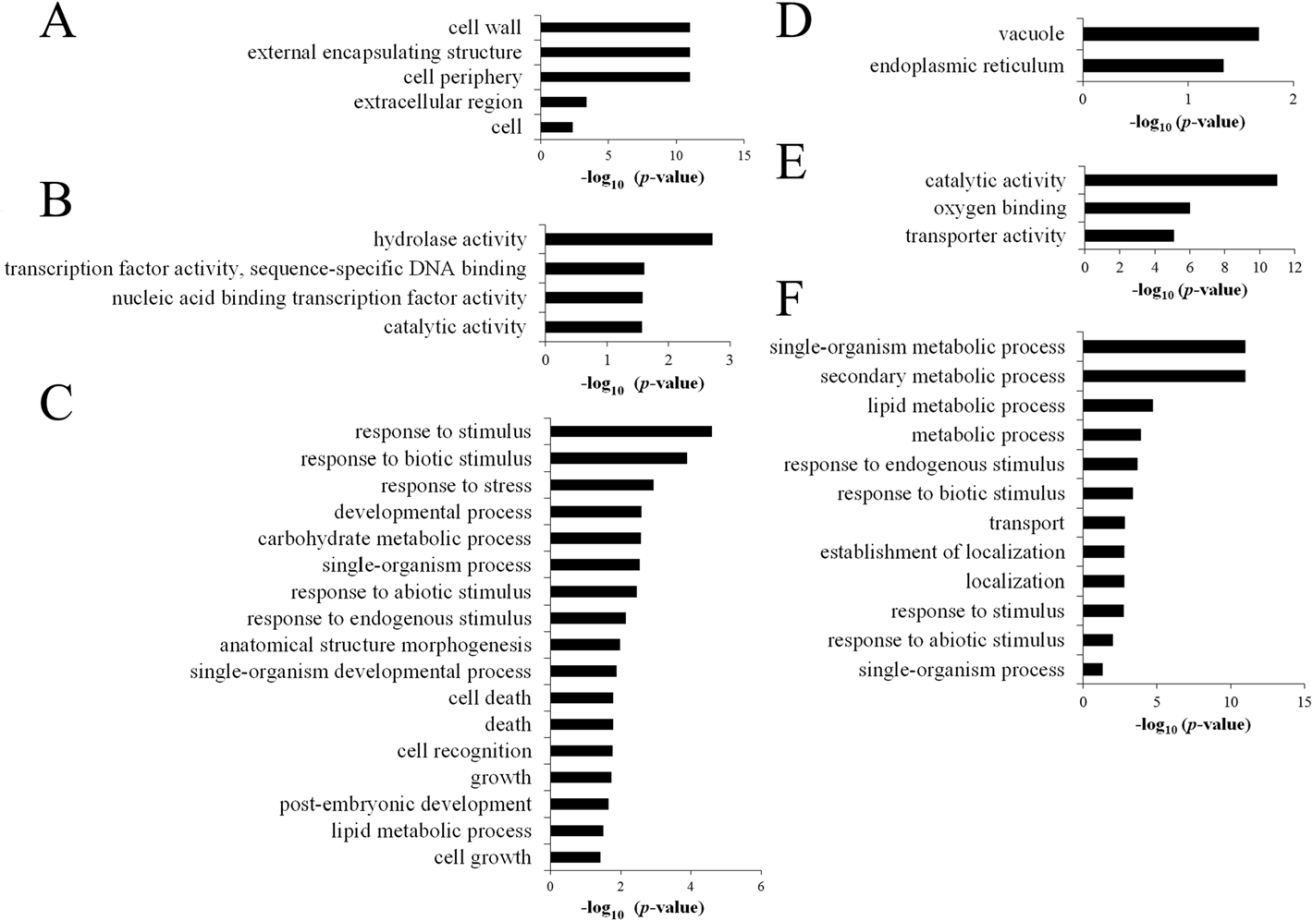
Gene Ontology (GO) analysis for differentially expressed genes (DEGs). **a**, Significant cellular component GO terms of the up-regulated DEGs. **b**, Significant molecular function GO terms of the up-regulated DEGs. **c**, Significant biological process GO terms of the up-regulated DEGs. **d**, Significant cellular component GO terms of the down-regulated DEGs. **e**, Significant molecular function GO terms of the down-regulated DEGs. **f**, Significant biological process GO terms of the down-regulated DEGs

To further investigate the regulatory mechanism of *REL1*-mediated leaf rolling and bending, we performed a RNA-seq analysis with the leaves of *rel1-D* mutants and wild type plants at the tillering stage, since the most obvious leaf rolling and bending phenotypes were occurred at this stage. In total, 487 differentially expressed genes (DEGs) were identified with the stringent criteria (|log_2_ (FC)| ≥ 1, and FDR < 0.05). To verify these DEGs, 10 randomly selected DEGs were detected by the qRT-PCR, and their trends were similar as RNA-seq (Additional file 4: Figure S2), indicating that the RNA-seq result was qualified for following study. Of these DEGs, 247 and 240 transcripts were up-regulated or down-regulated in *rel1-D* as compared to wild type, respectively (Additional file 5: Table S2). Even the statistical examination of most BR genes was not significant, there were still two BR signaling genes were induced in *rel-D* (Additional file 6: Figure S3), suggesting that BR was also involved in regulating the leaf morphology of *rel1-D*.Notably, the ABA pathway was significantly changed (Additional file 7: Figure S4), further supporting the note that ABA was involved in *rel1-D* mediated leaf morphology and response. To further characterize these DEGs, we performed the gene ontology (GO) enrichment analysis. In respect to the up-regulated DEGs, they were significantly assigned to certain cellular component GO terms, including cell wall, external encapsulating structure and cell periphery (Fig. 3a; Additional file 8: Table S3). In terms of the molecular function GO term, these DEGs were mainly associated with hydrolase activity, transcription factor activity and catalytic activity (Fig. 3b; Additional file 9: Table S4). In respect to the biological process GO term, these DEGs were significantly involved in multiple stimuli and stresses (Fig. 3c; Additional file 10: Table S5). Investigation of the down-regulated DEGs showed that they were also significantly associated with vacuole and endoplasmic reticulum (Fig. 3d; Additional file 11: Table S6), catalytic activity and transport activity (Fig. 3e; Additional file 12: Table S7), and response to various stimuli and metabolic processes (Fig. 3f; Additional file 13: Table S8). Taken together, our results suggested that boosting *REL1* apparently perturbed the homeostasis of stress dynamics in specific organelles, such as cell wall and vacuole, eventually leading to the abnormal leaf morphology.

### Proteomics analysis of *rel1-D* mutant

**Fig. 4:**
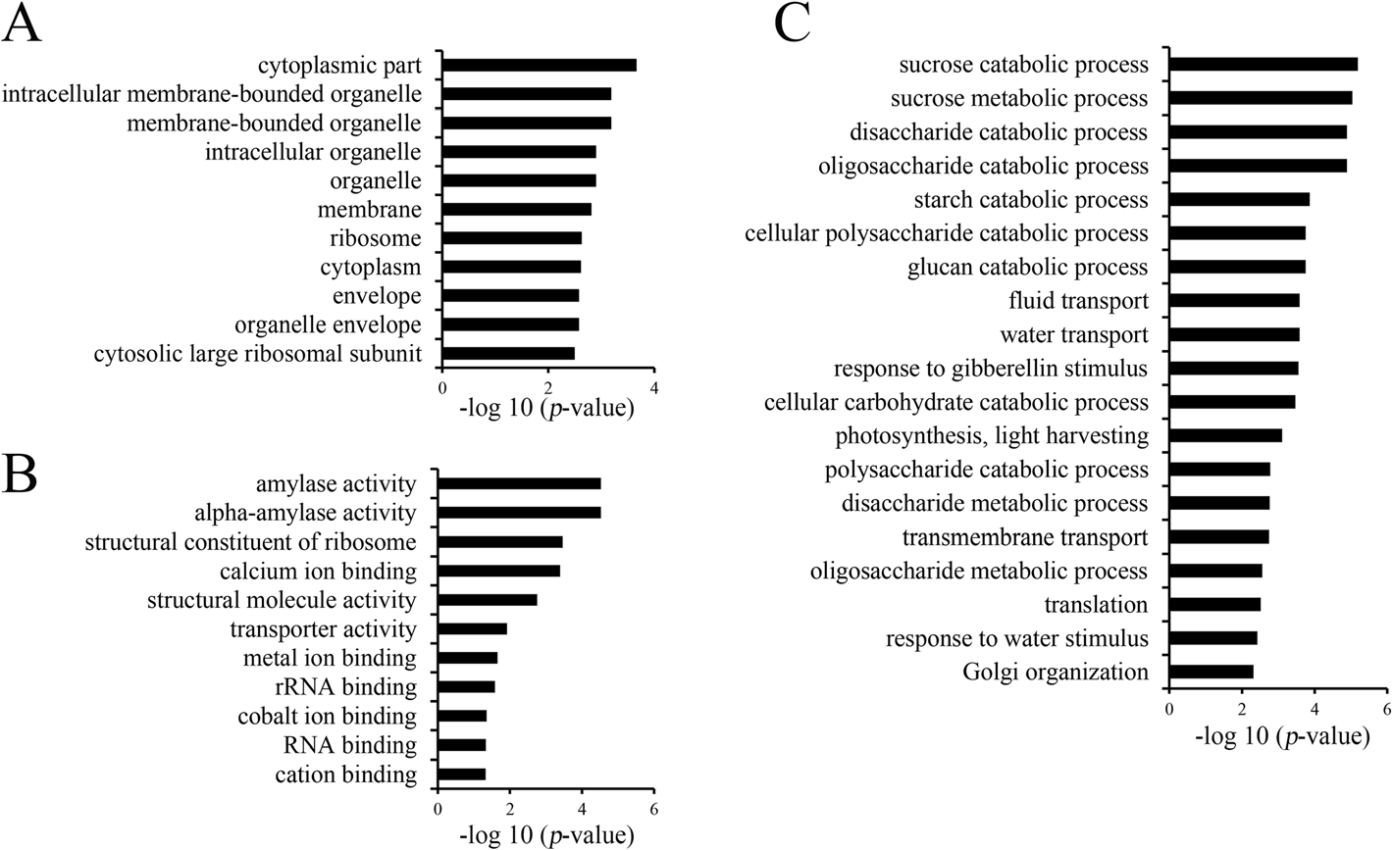
Gene Ontology (GO) analysis for differentially expressed proteins (DEPs). **a**, The 11 significant cellular component GO terms of the DEPs. **b**, The 11 molecular function GO terms of the DEPs. **c**, The 19 significant biological process GO terms of the DEPs

To further analyze the function of *REL1* in leaf morphology at protein level, we then performed the isobaric tags for relative and absolute quantitation (iTRAQ) analysis on the above materials. In total, 3657 peptides were identified (Additional file 14: Table S9). Using the *p*-value < 0.05 and fold change > 1.5 or < 0.67 as significant cutoff, 20 and 29 differentially expressed proteins (DEPs) were up-regulated or down-regulated, respectively. To verify these DEPs, 10 randomly selected DEPs were detected by the qRT-PCR, and their changing pattern were similar as iTRAQ (Additional file 15: Figure S5), indicating that the iTRAQ result was qualified for following study. Subsequently, GO enrichment analysis of these DEPs revealed that they were enriched for cellular component GO terms related to inter- and intra- cellular organelles, ribosome and plastid envelope (Fig. 4a; Additional file 16: Table S10). In respect to the molecular function GO term, significant enrichments were found in amylase activity, transport activity and ion binding (Fig. 4b; Additional file 16: Table S10). Regarding the biological process, the DEPs were grouped into multiple catabolic processes, response to water transport and stimuli (Fig. 4c; Additional file 16: Table S10). It was worthy to mention that 24 out of 49 DEPs were annotated or predicted to be plastid-localized proteins (Additional file 17: Table S11), while REL1 was previously implicated as plastid protein (Chen et al. 2015). Notably, 4 DEPs have been reported to regulate stress response (Table 1), including *OsPIP1;1*, *OsPIP1;2*, *SUS2* and *OsGLP8–7* (Liu et al. 2013; Mosa et al. 2012; Xiao et al. 2014; Breen and Bellgard 2010), further supporting the notion that *rel1-D* actives the endogenous stress responses. Collectively, we proposed that REL1 may coordinate the chloroplast DEPs to regulate leaf morphology by altering the metabolic process and stressful dynamics.

**Table 1:** The differentially expressed proteins (DEPs) related to the stress response

Gene locus	Symbol	Fold change^a^	Description (reference)
*LOC_Os02g44630*	*OsPIP1;1*	0.57 ± 0.13	Regulating the salt tolerance (Liu et al. 2013)
*LOC_Os04g47220*	*OsPIP1;2*	0.59 ± 0.02	Regulating the tolerance and transport of arsenate (Mosa et al. 2012)
*LOC_Os06g09450*	*SUS2*	0.58 ± 0.01	Induced by immerge during germination (Xiao et al. 2014)
*LOC_Os08g09010*	*OsGLP8–7*	1.62 ± 0.15	Induced by wounding and blast (Breen and Bellgard, 2010)

^a^The fold change was calculated by the *rel1-D* versus WT

### Integrative analysis of transcriptome and proteome for *rel1-D*

**Fig. 5:**
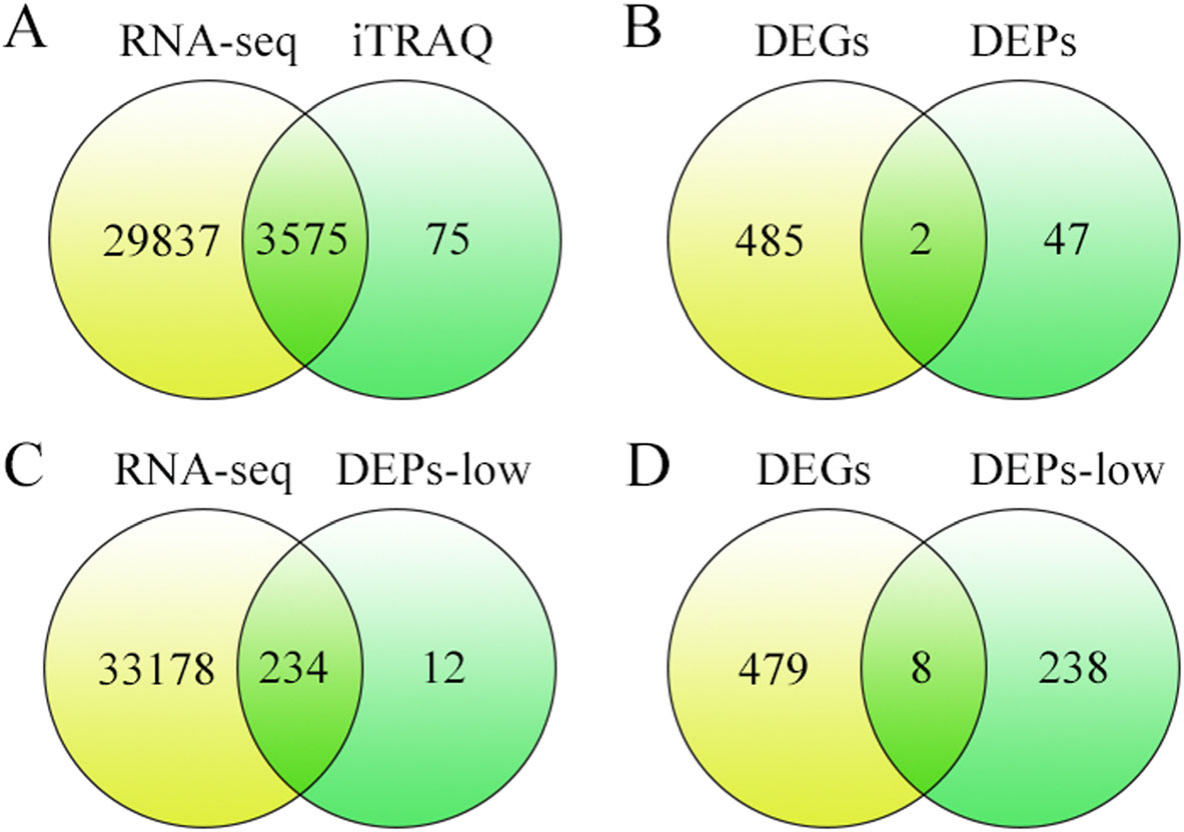
Correlation between the transcript and protein levels. **a**, Venn analysis of all expressed mRNA and all expressed proteins. **b**, Venn analysis of differentially expressed genes (DEGs) and differentially expressed proteins (DEPs) with stringent criteria (*p*-value < 005). **c**, Venn analysis of all expressed transcripts and differentially expressed proteins with less stringent criteria. **d**, Venn analysis of differentially expressed transcripts and differentially expressed proteins with less stringent criteria

Integrative analysis of transcriptome and proteome may provide new insights into the identification of interest key genes. In total, 3575 co-expressed genes and proteins were identified (Fig. 5a). Then only 2 co-expressed genes/proteins, *LOC_Os05g09740* and *LOC_Os02g37654*, were found between the 487 DEGs and 49 DEPs (Fig. 5b). To broad view the genome-wide change, 246 DEPs were screened by a less stringent criteria (with fold change > 1.5 or < 0.67), and 234 out of these low criteria DEPs (DEPs-low) were identified in the RNA-seq data (Fig. 5c). Integrating the DEGs and the 246 DEPs-low, there were 8 genes/proteins found in each other (Fig. 5d). These 8 genes modulated stresses response and had distinct expression pattern, of which 5 genes showed similar trends in transcription and translation level but 3 genes exhibited opposite trends (Table 2). Taken together, we proposed that the molecular mechanism underlying *REL1-*mediated leaf phenotype was likely different between transcriptional and post-translation levels.

**Table 2:** Expression pattern of DEGs and DEPs identified in both transcriptomic and proteomic analyses

Gene locus	Transcript^a^	Protein^b^	Description
*LOC_Os01g51570*	2.45 ± 1.36	1.58 ± 0.21	Glycosyl hydrolases family 17, putative, expressed
*LOC_Os08g39840*	1.19 ± 0.10	0.61 ± 0.10	*OsHI-LOX*; *RLL*; *OsLOX9*
*LOC_Os02g33820*	−1.05 ± 0.28	1.52 ± 0.42	*Asr3*; *OsASR3*; *ASR1*
*LOC_Os02g37654*	−2.02 ± 0.47	0.58 ± 0.11	Lecithin: cholesterol acyltransferase, putative, expressed
*LOC_Os03g40670*	1.20 ± 0.34	0.63 ± 0.10	Glycerophosphoryl diester phosphodiesterase family protein, putative, expressed
*LOC_Os05g09740*	−4.16 ± 0.65	0.51 ± 0.06	HAD superfamily phosphatase, putative, expressed
*LOC_Os05g10210*	−5.67 ± 0.31	0.62 ± 0.00	HAD superfamily phosphatase, putative, expressed
*LOC_Os10g36170*	−1.81 ± 0.81	0.52 ± 0.02	LTPL160 - Protease inhibitor/seed storage/LTP family protein precursor, expressed

^a^ Represent the fold change of the DEGs in RNA-seq analysis

^b^ Represent the fold change of DEPs in iTRAQ analysis

## Discussion

During plant growth and development, leaf rolling is an adaptive rather than passive response to the abiotic and biotic stresses in plants (Kadioglu et al. 2012; Kadioglu and Terzi, 2007). Our previous study has implicated that *REL1* positively regulates leaf rolling through altering the profile of bulliform cells and leaf bending by coordinated expression of BR related genes (Chen et al. 2015). However, the biological function of *REL1* and the relevant regulatory mechanism still remains to be further elucidated. Here, we further explored its regulatory role in leaf morphology by transcriptomic and proteomic analyses, as well as co-expression analysis. Our results may provide a new insight into the *REL1*-mediated leaf morphology in rice.

Advance studies have also been evident that water deficiency is one of the most major reasons for the formation of leaf rolling, which effectively reduces transpiration and thus is potentially useful in drought tolerance (Kadioglu et al. 2012). As expected, up-regulation of drought resistance marker genes and tolerance to drought treatment in *rel1-D* suggested that *REL1* positively participates in drought resistance. In addition, our result also indicated that *REL1*-mediated drought tolerance might be integrated the ABA pathway. Therefore, we proposed that *REL1* might coordinate ABA pathway to regulate drought tolerance. Comparative analysis of *REL1* and other abaxial leaf rolling genes demonstrated that they might play opposite roles in response to drought tolerance, suggesting that they might also function independently during the formation of leaf rolling under stresses. Surprisingly, we also found that *REL1* is involved in leaf senescence and ABA response, which would be another interesting issue to be addressed regarding the biological function of *REL1*.

Although *REL1* encodes an unknown but species-specific protein, genome-wide profiling may facilitate our understanding on the biological function of *REL1* in determining leaf morphology. Transcriptomic and proteomic profiling demonstrated that the change of leaf morphology in *rel1-D* was highly associated with the metabolic changes and stresses response. However, it is still unclear whether *REL1* directly or indirectly catalyzes specific primary and/or secondary metabolism so that generating stressful dynamics endogenously. Taking into account the chloroplast localization of REL1, the chloroplast DEGs may be functionally associated with *REL1*. Unexpectedly, only 4 DEGs were grouped into chloroplast GO term under the stringent statistical criteria. Differing from the DEGs, almost half of the DEPs were chloroplast localized proteins. Combining the integrative analyses of transcriptom and proteasome, two possibilities were proposed: 1) *REL1* regulates leaf morphology at the post-transcriptional level independent on the chloroplast genes; 2) *REL1* regulates leaf morphology at the post-translation levels through direct or indirect regulation of chloroplast proteins. These issues would be quite interesting for further study. Expectedly, a large part of the DEGs and DEPs were both enriched into the stress response GO term, further demonstrating that up-regulation of *REL1* generates endogenous stresses for the plant. Several genes/proteins identified in both DEGs and DEPs would be the candidates for future study in terms of leaf rolling and bending, particularly the response to multiple stresses.

Interestingly, a recent study reported that another gene *REL2*, encoding a DUF630 and DUF632 domains containing protein, regulates leaf rolling and bending as well (Yang et al. 2016). It was worthy to figure out that the gene *LOC_Os06g44610*, significantly down-regulated in *rel1-D*, also encodes a membrane associated DUF588 domain containing protein, suggesting a possible role of DUF family genes in the leaf development. Although *REL1* has a functional relationship with *REL2*, it is still challenged by: 1) the distinct localization pattern of these two proteins since REL2 is a plasma membrane localized protein while REL1 is a chloroplast protein; 2) *REL2* likely regulates bulliform cell through auxin pathway while *REL1*might be much more related to BR pathway. In addition, much more transcriptomics datasets would benefit the further understanding of the regulatory network between *REL1* and other leaf development genes by co-expression analysis. Meanwhile, genetic analysis by constructing double (or multiple) mutant would also benefit our knowledge of the correlation among these leaf morphology genes.

## Conclusions

Leaf rolling is generally related to stress response. By the drought assay, we identified the involvement of *rel1-D* in such biological process. Furthermore, the RNA-seq and iTRAQ-based profiling of WT and *rel1-D* also indicated that enhanced expression of *REL1* resulted in the alteration of stress responsive genes and hormone genes. These genes might be the potential targets for extending our understanding of the *REL1*-mediated leaf morphology, and would also be valuable resources for further exploring and/or genetic engineering the molecular mechanism of stress tolerance in rice.

### One sentence summary

*REL1* is involved in drought tolerance, leaf senescence and hormones sensitivity by altering the profiles of relevant response genes in rice.

## Additional files

Additional file 1: **Table S12.** Primers used in this study. (XLSX 12 kb)

Additional file 2: **Table S1.** Expression of drought-responsive genes in WT and *rel1-D*. (XLSX 13 kb)

Additional file 3: **Figure S1.** Expression of abaxial leaf rolling genes in response to PEG treatment. **a** Expression of *REL1* during different time courses by PEG treatment. **b** Expression of *ACL1* during different time courses by PEG treatment. **c** Expression of *ACL2* during different time courses by PEG treatment. **d** Expression of *Roc5* during different time courses by PEG treatment. **a**-**d** Multiple comparisons, Duncan, *p*-value < 0.01. (TIF 2233 kb)

Additional file 4: **Figure S2.** Verification of the RNA-seq. Modes for qPCR were cDNA of WT and the *rel1-D* leaves in tillering stage, three biological repetitions, error bars were S.E.; the X axis was gene names, the Y axis was log_2_ (Fold change). (TIF 56 kb)

Additional file 5: **Table S2.** Differentially expressed genes in *rel1-D* mutant. (XLSX 190 kb)

Additional file 6: **Figure S3.** Expressions of BR-related genes. A Expressions of 10 BR synthesis-related genes. B Expressions of 31 BR signaling genes. A-B The colors indicated the mean expression level (FPKM), the red color was the highest and the green color was the lowest; red panes highlighted *p*-value < 0.05. (TIF 87 kb)

Additional file 7: **Figure S4.** Expressions of ABA-related genes. A Expressions of 15 ABA synthesis-related genes (positive-related). B Expressions of 3 ABA synthesis-related genes (negative-related). C Expressions of 43 ABA signaling genes. A-C The colors indicated the mean expression level (FPKM), the red color was the highest and the green color was the lowest; red panes highlighted *p*-value < 0.05. (TIF 141 kb)

Additional file 8: **Table S3.** GO enrichment (Cellular Component) of the up-regulated DEGs in *rel1-D*. (XLSX 121 kb)

Additional file 9: **Table S4.** GO enrichment (Molecular Function) of the up-regulated DEGs in *rel1-D*. (XLSX 121 kb)

Additional file 10: **Table S5.** GO enrichment (Biological Process) of the up-regulated DEGs in *rel1-D*. (XLSX 126 kb)

Additional file 11: **Table S6.** GO enrichment (Cellular Component) of the down-regulated DEGs in *rel1-D*. (XLSX 121 kb)

Additional file 12: **Table S7.** GO enrichment (Molecular Function) of the down-regulated DEGs in *rel1-D*. (XLSX 121 kb)

Additional file 13: **Table S8.** GO enrichment (Biological Process) of the down-regulated DEGs in *rel1-D*. (XLSX 125 kb)

Additional file 14: **Table S9.** Peptides identified in iTRAQ. (XLSX 3162 kb)

Additional file 15 **Figure S5.** Verification of the iTRAQ. Modes for qPCR were cDNA of WT and the *rel1-D* leaves in tillering stage, three biological repetitions, error bars were S.E.; the X axis was gene names, the Y axis was log_2_ (Fold change of transcript). (TIF 41 kb)

Additional file 16: **Table S10.** Plastid associated differentially expressed proteins in *rel1-D*. (XLSX 13 kb)

Additional file 17: **Table S11.** Primers used in this study. (XLSX 12 kb)
