# Supplementary figures and images for: Constitutive expression of REL1 confers the rice response to drought stress and abscisic acid
Source: Rice (N Y). 2018 Oct 25;11:59. doi: 10.1186/s12284-018-0251-0 (PMC6202306; doi:10.1186/s12284-018-0251-0)

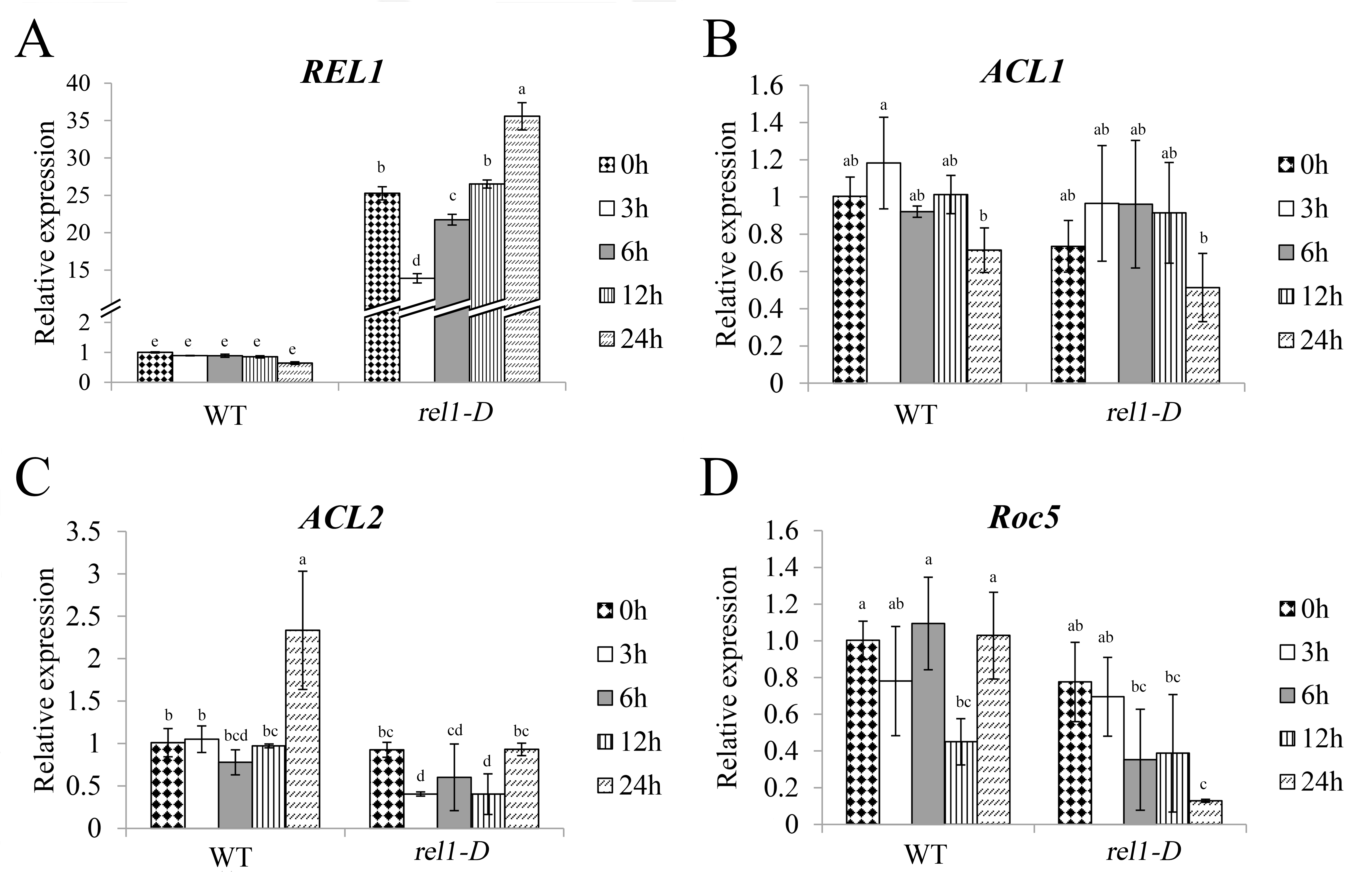

Supplement: Supplementary file 3 — Figure S1. Expression of abaxial leaf rolling genes in response to PEG treatment. a Expression of REL1 during different time courses by PEG treatment. b Expression of ACL1 during different time courses by PEG treatment. c Expression of ACL2 during different time courses by PEG treatment. d Expression of Roc5 during different time courses by PEG treatment. a-d Multiple comparisons, Duncan, p-value < 0.01. (TIF 2233 kb) [file 12284_2018_251_MOESM3_ESM.tif]

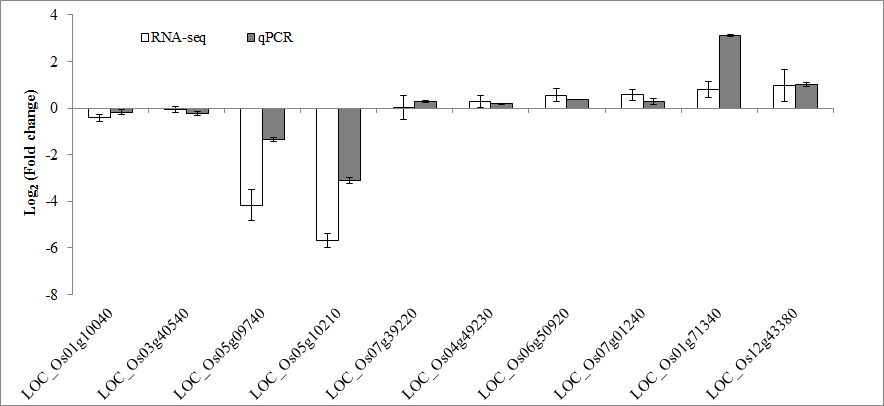

Supplement: Supplementary file 4 — Figure S2. Verification of the RNA-seq. Modes for qPCR were cDNA of WT and the rel1-D leaves in tillering stage, three biological repetitions, error bars were S.E.; the X axis was gene names, the Y axis was log2 (Fold change). (TIF 56 kb) [file 12284_2018_251_MOESM4_ESM.tif]

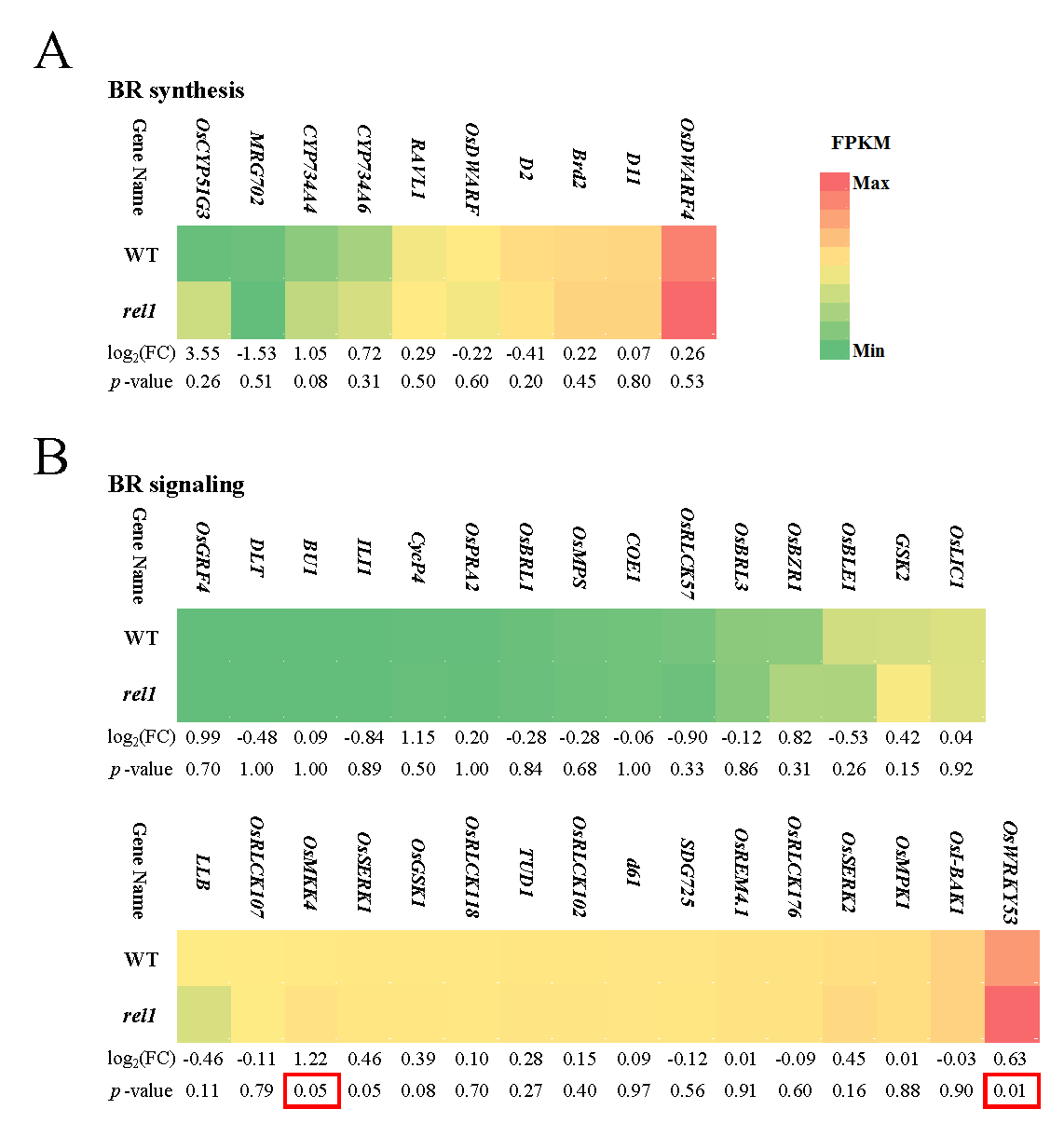

Supplement: Supplementary file 6 — Figure S3. Expressions of BR-related genes. A Expressions of 10 BR synthesis-related genes. B Expressions of 31 BR signaling genes. A-B The colors indicated the mean expression level (FPKM), the red color was the highest and the green color was the lowest; red panes highlighted p-value < 0.05. (TIF 87 kb) [file 12284_2018_251_MOESM6_ESM.tif]

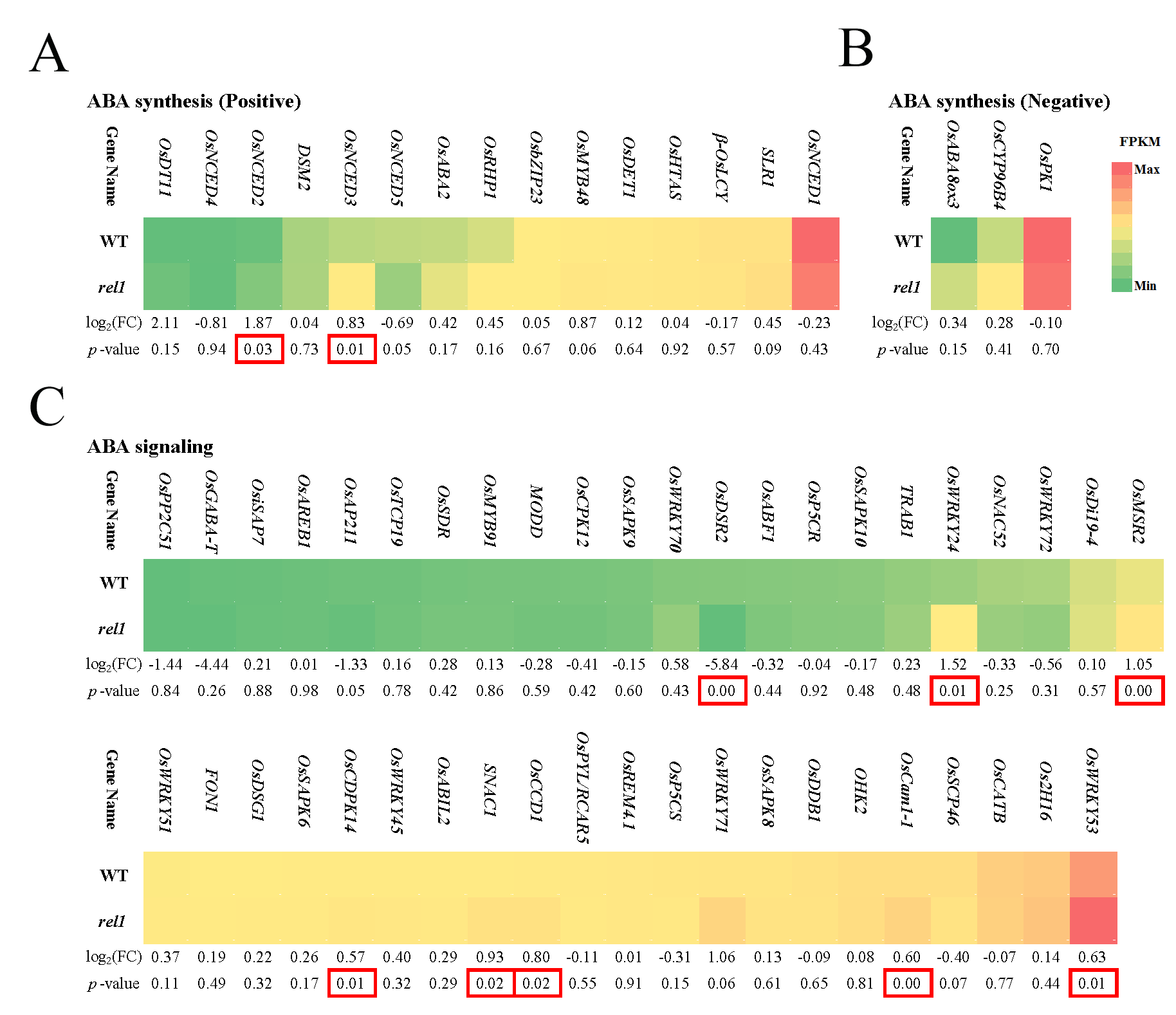

Supplement: Supplementary file 7 — Figure S4. Expressions of ABA-related genes. A Expressions of 15 ABA synthesis-related genes (positive-related). B Expressions of 3 ABA synthesis-related genes (negative-related). C Expressions of 43 ABA signaling genes. A-C The colors indicated the mean expression level (FPKM), the red color was the highest and the green color was the lowest; red panes highlighted p-value < 0.05. (TIF 141 kb) [file 12284_2018_251_MOESM7_ESM.tif]

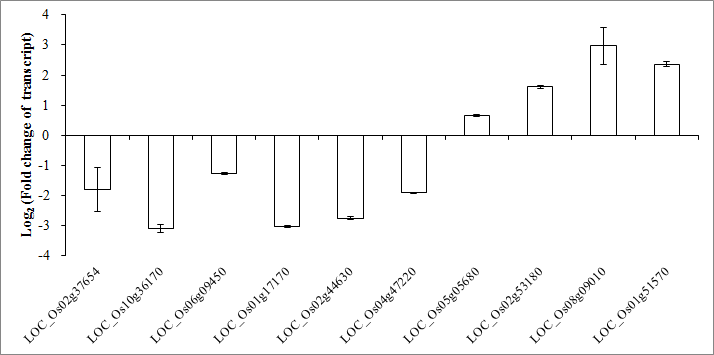

Supplement: Supplementary file 15 — Figure S5. Verification of the iTRAQ. Modes for qPCR were cDNA of WT and the rel1-D leaves in tillering stage, three biological repetitions, error bars were S.E.; the X axis was gene names, the Y axis was log2 (Fold change of transcript). (TIF 41 kb) [file 12284_2018_251_MOESM15_ESM.tif]
